# Routine Immunohistochemical Analysis of Mismatch Repair Proteins in Colorectal Cancer—A Prospective Analysis

**DOI:** 10.3390/cancers14153730

**Published:** 2022-07-31

**Authors:** Joana Lemos Garcia, Isadora Rosa, Sofia Saraiva, Inês Marques, Ricardo Fonseca, Pedro Lage, Inês Francisco, Patrícia Silva, Bruno Filipe, Cristina Albuquerque, Isabel Claro

**Affiliations:** 1 Gastroenterology Department, Instituto Português de Oncologia de Lisboa Francisco Gentil, 1099-023 Lisbon, Portugal; isarosa@ipolisboa.min-saude.pt (I.R.); amenezes@ipolisboa.min-saude.pt (S.S.); inesnmarques3@gmail.com (I.M.); plage@ipolisboa.min-saude.pt (P.L.); iclaro@ipolisboa.min-saude.pt (I.C.); 2 Familial Cancer Clinic, Instituto Português de Oncologia de Lisboa Francisco Gentil, 1099-023 Lisbon, Portugal; mfrancisco@ipolisboa.min-saude.pt (I.F.); palsilva@ipolisboa.min-saude.pt (P.S.); bfilipe@ipolisboa.min-saude.pt (B.F.); calbuque@ipolisboa.min-saude.pt (C.A.); 3 Pathology Department, Instituto Português de Oncologia de Lisboa Francisco Gentil, 1099-023 Lisbon, Portugal; rifonseca@ipolisboa.min-saude.pt; 4 Molecular Pathobiology Investigation Unit, Instituto Português de Oncologia de Lisboa Francisco Gentil, 1099-023 Lisbon, Portugal

**Keywords:** colorectal cancer, Lynch Syndrome, mismatch repair proteins

## Abstract

**Simple Summary:**

Recognition of a hereditary colorectal cancer (CRC) syndrome is crucial. Our aim was to assess the value of routine immunohistochemistry screening for mismatch repair proteins deficiency in CRC patients under 70 years-old. In our cohort, this inclusive strategy allowed the identification of Lynch Syndrome patients that could otherwise be missed using a restrictive approach that relies only on Amsterdam and Bethesda criteria. This study strengthens current recommendations and highlights the role of universal CRC screening for MMR protein status.

**Abstract:**

Recognition of a hereditary colorectal cancer (CRC) syndrome is crucial and Lynch Syndrome (LS) is the most frequent immunohistochemistry (IHC)—screening for mismatch repair proteins (MMR) deficiency in CRC is therefore advocated. An unicentric cohort study was conducted in a central Oncological Hospital to assess its results. All patients under 70 years-old admitted between July 2017–June 2019 and submitted to surgery for CRC were included. Of 275 patients, 56.0% were male, median age 61.0 (IQR:54.5–65.0), with synchronous tumors in six. Histology revealed high grade adenocarcinoma in 8.4%; mucinous and/or signet ring differentiation in 11.3%; and lymphocytic infiltration in 29.8%. Amsterdam (AC) and Bethesda (BC) Criteria were fulfilled in 11 and 74 patients, respectively. IHC revealed loss of expression of MMR proteins in 24 (8.7%), mostly MLH1 and PMS2 (*n* = 15) and PMS2 (*n* = 4). Among these, no patients fulfilled AC and 13 fulfilled BC. BRAF mutation or MLH1 promoter hypermethylation was found in four patients with MLH1 loss of expression. Genetic diagnosis was performed in 51 patients, 11 of them with altered IHC. LS was diagnosed in four, and BC was present in three. One patient would not have been diagnosed without routine IHC screening. These results strengthen the important role of IHC screening for MMR proteins loss of expression in CRC.

## 1. Introduction

Colorectal cancer (CRC) is the third most common cancer type [1,2,3] and its incidence in some developed countries is increasing among the young (less than 50 years-old) [4,5,6,7,8]. Hereditary syndromes may be responsible for 15–22% of CRC cases [7,9,10].

Recognition of a hereditary CRC syndrome is of paramount importance, since it impacts on patients’ surgical management and surveillance as well as on their families screening and surveillance programs [11]. Lynch Syndrome (LS) is the most frequent hereditary CRC syndrome, accounting for 1–3% of all CRC. It occurs due to autosomal dominant mutations in the mismatch repair (MMR) genes *MLH1*, *MSH2*, *MSH6* and *PMS2* or deletions on the cell adhesion molecule (*EPCAM*) gene, which is located upstream of *MSH2*. The MMR defect (which may also be somatic, mostly due to MLH1 promoter hypermethylation) will lead to failure to correct DNA replication errors with accumulation of mutations, resulting in a microsatellite instability (MSI) phenotype. Diagnosis of MSI is via polymerase chain reaction (PCR) amplification of specific microsatellite repeats. Alternatively, immunohistochemistry (IHC) can show absence of expression of MMR proteins in the tumor [12].

Lynch Syndrome can be suspected through family history and clinical data collection, considering the Amsterdam criteria and the revised Bethesda guidelines (Table 1), or using computer-based calculators [12]. However, this strategy lacks sensitivity and specificity. Clinical criteria limitations are overcome by routine IHC staining for MMR proteins in all CRC samples [13,14,15] in a cost-effective manner [16,17,18]. International guidelines recommend tumor screening for MMR deficiency for all colorectal cancers regardless of age at diagnosis [19,20] or in patients bellow 70 years-old [21]. In case of MSH2, MSH6 or PMS2 loss of expression, germline testing should ensue. If there is loss of MLH1 or MLH1/PMS2 expression, somatic tumor mutations should be ruled-out first, by searching for *BRAF V600E* mutation and/or *MLH1* promoter hypermethylation [12].

Currently, MMR defects’ identification in CRC and has a role beyond LS identification—selection of stage II patients for chemotherapy (CT), choice of the type of adjuvant CT and selection of stage IV patients for immunotherapy all depend on MSI status.

The goal of this study was to assess the importance of routine IHC screening for MMR defects in CRC patients in the identification of Lynch Syndrome patients, in a real-world setting.

## 2. Materials and Methods

A unicentric cohort study was conducted at the Portuguese Oncological Institute of Lisbon, Portugal, which integrates a Familial Risk Clinic. In this hospital, around 290 new colorectal cancer patients are admitted per year by the Multidisciplinary Colorectal Cancer Group. In their first appointment, relevant personal and clinical data are collected, including family history of neoplasia. All CRC cases are reviewed in a weekly multidisciplinary meeting. All tumors are classified according to the World Health Organization (WHO) Classification of Tumors (2019) [24] and staged using the American Joint Committee on Cancer (AJCC) (8th edition) [25] TNM system.

### 2.1. Patient Selection

All patients reviewed in the multidisciplinary CRC meeting from 01-07-2016 to 30-06-2019 who were 70 years-old or younger and underwent primary tumor resection surgery were included, in a total of 275 patients.

### 2.2. Data Collection

Data collected included demographic information, tumor location, radiological and pathological staging, therapeutic modalities performed, family history of CRC and other LS-spectrum cancers, MMR protein status, *BRAF V600E* mutation status, MMR gene promotor methylation and germline mutation analysis. For stage at diagnosis classification, pathological staging was the gold standard, except in patients who underwent neoadjuvant treatment, for whom radiological staging at diagnosis was preferred.

### 2.3. Hospital Standard Procedures

#### 2.3.1. CRC Sample Processing

**Figure 1:**
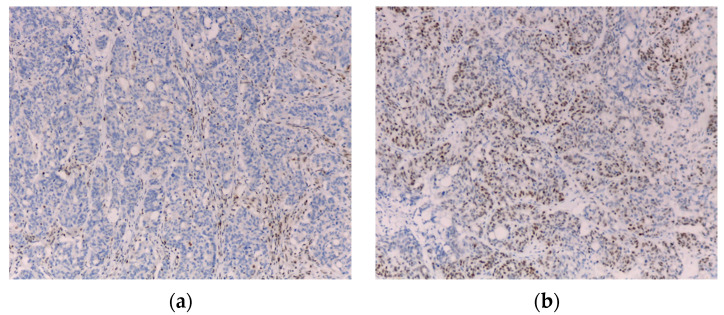
Immunohistochemistry showing loss of MLH1 (**a**) and maintained MSH2 (**b**) staining (10×).

In our institution, until 2021, according to the 2009 Jerusalem Workshop recommendations [21], in all patients 70 years old or younger who underwent surgery for CRC, the tumor was screened for loss of expression of MMR proteins by immunohistochemistry. To assess the expression of MLH1, PMS2, MSH2 and MSH6 proteins, IHC analysis is performed using Ventana CC1 equipment (sample in 10% formalin buffer, using thermal recuperation method) and monoclonal antibodies anti-MLH1 (clone ES05), anti-PMS2 (clone EP51), anti-MSH2 (clone G219-1129) and anti-MSH6 (clone EP49) (Figure 1).

To exclude somatic mutations that lead to MLH1-defective cases, since 2019, tumors with MLH1 loss of expression are further investigated for *BRAF V600E* mutation: DNA from samples of tumor tissue is amplified by PCR using primers for BRAF exon 15 and the product is sequenced using Sanger sequencing on Big Dye terminator v1.1 sequencing kit (Applied Biosystems) on an automatic ABI PrismTM 3130 Genetic Analyzer (Applied Biosystems).

BRAF V600E mutation analysis results were also available in some stage IV (at diagnosis or during follow-up) patients, in whom the test was performed for chemotherapy selection, regardless of IHC results.

#### 2.3.2. Family Risk Clinic Referral

In case Amsterdam or revised Bethesda criteria are fulfilled or when germline MMR genes’ mutations are suspected after IHC analysis, the patients are referred to the Familial Risk Clinic. All patients with 10 or more adenomas or those who fulfil the World Health Organization criteria for Serrated Polyposis Syndrome are also referred.

In cases referred for evaluation in the Familial Risk Clinic, additional tumor testing before genetic diagnosis may be done, at physician’s discretion, according to available evidence and international recommendations.

#### 2.3.3. Molecular and Genetic Testing

##### Microsatellite Instability Analysis

Between 2016 and 2017, this was carried out using the Bethesda microsatellite markers: BAT26, BAT25, D17S250, D2S123 and D5S346 [26,27,28]. In tumor samples exhibiting microsatellite instability (MSI) in only one marker, or without a conclusive result in at least one marker, two additional markers were analyzed (BAT40 and MYCL1). From 2017 onwards, the MSI analysis was performed with 10 microsatellite markers (the above mentioned and 3 additional mononucleotide repeat marker—NR21, NR24 and NR27).

Between 2016 and 2017, DNA was isolated from CRC-PDEs samples using the KAPA Express Extract Kit (KAPABIOSYSTEMS, Potters Bar, United Kingdom) and from paraffin-embedded tissue (FFPE) colorectal cancer and normal colonic mucosa using proteinase K digestion, which was followed by phenol/chloroform extraction and ethanol precipitation [29]. From 2017, the Maxwell^®^ RSC DNA FFPE Kit (Promega, Madison, WI, USA) was used to isolate DNA from FFPE samples in the Maxwell^®^ RSC Instrument (Promega). Each tumor and paired normal DNA were amplified by PCR for each of the microsatellite markers, using fluorescent labelled primers (Applied Biosystems, Foster City, CA USA), specific for each locus [30,31]. PCR products were analyzed in the ABI PrismTM 3130 Genetic Analyzer using the GeneMapper software (Applied Biosystems). Tumors presenting MSI in >40% of the markers analyzed were classified as MSI-High (MSI-H); otherwise they were classified as MSI-Low (MSI-L) [32]. Tumors without MSI in any of the markers were considered to be microsatellite stable (MSS).

##### MMR Gene Promoter Methylation Analysis

The analysis of MMR gene promoters methylation was performed by methylation-specific multiplex ligation-dependent probe amplification (MS_MLPA) [33], using the MS-MLPA kits ME011 MMR (MRC-Holland, Amsterdam, the Netherlands). MS-MLPA reactions were performed as described by the manufacturer. MS-MLPA fragments were analyzed on the ABI Prism 3130TM Genetic Analyzer (Applied Biosystems) and normalized using the Coffalyser. NET software (MRC-Holland, Amsterdam, the Netherlands). A baseline for positive methylation was calculated for each gene as described previously [34]. A ratio of 0.15 or higher, corresponding to 15% of methylated DNA, was indicative of *MLH1* promoter methylation.

##### Germline Mutation Analysis

In case of MMR proteins’ deficiency in IHC analysis, mutations in *MLH1*, *MSH2*, *MSH6*, *PMS2* and *EPCAM* were investigated. In other cases, Next Generation Sequencing (NGS) multigene panels were used, according to clinical data and family history.

Germline mutation analysis was performed after signed informed consent, by NGS using multigene panels (TruSight Cancer kit (Illumina, San Diego, CA, USA)) and MLPA (multiplex ligation-dependent probe amplification) analysis (MRC-Holland, Amsterdam, the Netherlands). All pathogenic, probably pathogenic or of uncertain pathogenicity mutations (frequency less than 1% in the population) are confirmed by Sanger sequencing, from an independent DNA sample. The interpretation of the variants is performed according to the rules established by LOVD-InSIGHT (International Society for Gastrointestinal Hereditary Tumors—http://www.insight-group.org/criteria last accessed on the 1 June 2022).

### 2.4. Statistical Analysis

For statistical analysis, SPSS Statistics 26 (IBM) was used. Demographic and clinical characteristics were presented as frequencies. Continuous variables were expressed as median and standard deviation or as median and interquartile range, according to data distribution, and were compared using t-Student or Wilcoxon tests, respectively. Qualitative variables were compared using chi-square or Fisher Exact tests. Multiple variables were analyzed using logistic regression models. A *p* value lower than 0.05 was considered statistically significant.

## 3. Results

### 3.1. Clinical Characterization

A total of 275 patients were included, 56.0% males, with a median age at diagnosis of 61.0 (IQR 54.5–65.0) years old. Tumors were mostly (53.1%) stage III at diagnosis and histological report revealed high grade (G3) tumors in 8.4%, mucinous and/or signed ring morphology in 11.3% and lymphocytic infiltrate in 29.8%. Population and tumor characteristics are depicted in Table 2 and Table 3. Mean follow-up time was 40.6 ± 15.6 months. After personal and family history investigation, 11 (4.0%) patients fulfilled Amsterdam criteria (AC) and 74 (26.9%) revised Bethesda criteria (BC).

### 3.2. Immunohistochemical Analysis

IHC evaluation revealed loss of MMR proteins’ expression in 24 cases (8.7%)– MLH1 and PMS2 (*n* = 15) (Figure 1); PMS2 (*n* = 4); MSH2 and MSH6 (*n* = 1); MSH2 (*n* = 1); MSH6 (*n* = 2); MLH1, PMS2 and MSH6 (*n* = 1). AC and BC were fulfilled in 0 and 13 of such cases, respectively (Table 4).

Altered IHC analysis showed a significant association with tumor location in the right colon (*p <* 0.001), poor differentiation (*p =* 0.015) and mucinous histology (*p =* 0.016), but not with gender (*p =* 0.157), age (*p =* 0.709), stage (*p =* 0.44), lympho-vascular (*p* = 0.279) or perineural invasion (*p =* 0.567), lymphocytic infiltrate (*p =* 0.052) or tumor budding (*p =* 0.499).

### 3.3. Analysis of MMR Deficient Cases—BRAFV600E Mutation Status, MMR Gene Methylation and Germline Mutation Analysis

From the 16 patients with MLH1 loss of expression (15 with MLH1/PMS2 loss of expression, one with MLH1/PMS2/MSH6 loss of expression), somatic BRAF V600E mutation testing was carried out in seven, and found in one patient—the IHC alteration was considered somatic and the patient was not referred for genetic testing. From the remaining six patients, three had MLH1 promoter hypermethylation and three did not show either of the somatic alterations. Genetic testing was performed in these last three patients, of whom one had confirmed LS; in the other two, no germline mutation was detected (Table 4).

BRAF V600E mutation testing results were also available in three other patients in whom the analysis was requested by oncologists, for chemotherapy selection (Table 4).

Five patients with altered IHC died before the Family Risk Clinic appointment/germline mutation analysis and one refused genetic testing. Family Risk Clinic appointment is pending or genetic testing is still ongoing in six patients.

Therefore, in total, genetic test results were available in 11 of the 24 patients with altered IHC and in one with artifacts, and Lynch Syndrome was diagnosed in four of them.

Patients with Lynch Syndrome were men in three cases, and aged less than 50 years-old in three (median age 37.0 (IQR 27.5–51.8)). AC were not fulfilled in any of the patients, and three met BC; IHC was altered in three and unavailable in one due to artifacts (Table 4).

Tumor was in the right colon in three and rectum in one, stage I in one and III in three cases. Histology report revealed low-grade (G1/G2) tumors with no other specification, no lymphocytic infiltrate and no unfavorable invasions in all cases.

All patients were alive without evidence of cancer relapse at last follow-up (median follow-up = 33.0 months (IQR: 26.8–54.3)).

The presence of Lynch Syndrome had a significant association with younger age at diagnosis (*p <* 0.001) and right-sided tumors (*p* = 0.037), but not with gender (*p =* 0.634), stage (*p =* 0.718), differentiation (*p =* 1.000), histological subtype (*p =* 1.000), lympho-vascular invasion (*p =* 0.575), perineural invasion (*p =* 1.000), lymphocytic infiltrate (*p =* 0.323) or tumor budding (*p =* 1.000).

### 3.4. Germline Mutation Analysis in MMR Proficient Cases

In 10 patients with altered IHC and in one with artifacts, germline MMR mutation analysis was performed and in 40 patients a multigene panel was used. From these, one Familial Adenomatous Polyposis and one *MUTYH*-associated Polyposis were diagnosed (both in patients with multiple adenomas). A *MUTYH* heterozygote mutation was found in a patient with CRC at the age of 47 with family history of colonic adenomas. Familial Colorectal Cancer Type X) was diagnosed in a patient in whom no mutation was found after multigene panel testing.

## 4. Discussion

This study presents the clinical picture of CRC in an adult population under 70 years old. As expected, most cases were sporadic cancers. Nevertheless, the use of IHC, combined with personal and familial data, allowed the attending physicians to diagnose Lynch Syndrome in four (1.5%) cases. It is important to notice that one of these patients did not fulfill Amsterdam II or Bethesda criteria and genetic diagnosis would have been missed if IHC analysis had not been performed.

Accurate and timely identification of Lynch Syndrome patients is extremely important, since surveillance for colonic and extra-colonic malignancies can increase survival and improve quality of live. This is relevant both for the patients and for at-risk relatives that may benefit from genetic study [13,35,36]. Even if LS is a rare entity, the cost of missing this diagnosis is significant.

Altered IHC was detected in 9.6% of the cases, a rate that is lower than expected, given that deficient-MMR protein status can be found in 15–30% of sporadic CRC [37]. The rates found may be due to the young population studied, where all CRC in patients aged more than 70 years old were excluded. Indeed, microsatellite instability in sporadic cases is frequently associated to MLH1 promoter methylation and these features are more frequently detected in older female patients, some of them often older than 70 years old [38].

In 16 patients, there was MLH1 ± PMS2 loss of expression in the tumor. A major limitation of our study was the fact that somatic *BRAF V600E* mutation/MLH1 promoter hypermethylation analysis’ results were available in only a minority of these cases. Routine *BRAF* testing after a MLH1 loss of expression result has only been implemented in our hospital in the last year of the study. Nevertheless, from seven patients with available results, one had *BRAF V600E* mutation and three others had *MLH1* promoter hypermethylation. These findings highlight the benefits of a step-up approach [20,39], that prevents a significant proportion of patients from undergoing most likely inconclusive genetic testing. This strategy makes sense not only in an economic standpoint, but also considering the psychological burden associated with genetic testing [39].

Further advantages of MMR status investigation are the possibility of personalized therapies. MSI tumors may have a reduced response to 5-FU chemotherapy and a better overall prognosis in early stages. Therefore, most stage II MMR deficient CRC patients do not seem to benefit from adjuvant chemotherapy, namely, with 5-FU [40]. Another scenario is metastatic MSI CRC, where therapy with immune checkpoint inhibitors may be proposed, since these patients often show sustained responses to this class of drugs. This is explained by the increased expression of several immune checkpoints in MMR deficient tumors, resulting from the production of abnormal proteins which elicit antigen-driven immune responses [40,41].

Although IHC analysis, molecular and genetic studies’ results were prospectively recorded, clinical data collection was retrospective, which is a limitation of the study, which may be relevant in details such as family history that may not have been carefully reported in all cases. However, the study was conducted in an oncological center which integrates a Family RiskClinic, in strict interaction with a Molecular Biology Laboratory and therefore has the means and expertise to pursue genetic investigation when indicated, limiting bias due to unrecognized hereditary cancer patients. Furthermore, this is a sequential series of patients with a relevant number of cases included, reflecting real-life practice.

## 5. Conclusions

This study strengthens current recommendations and highlights the role of universal CRC screening for MMR protein status. This inclusive strategy allows the identification of Lynch Syndrome patients that could otherwise be missed using a restrictive approach that relies only on Amsterdam and Bethesda criteria.

## Figures and Tables

**Table 1:** Clinical Criteria for Lynch Syndrome Screening (adapted from [22,23]).

**Amsterdam II**
At least 3 relatives with an HNPCC—associated cancer (CRC, endometrial, stomach, ovary, ureter/renal pelvis, brain, small bowel, hepatobiliary tract and skin (sebaceous) tumors) 1. One is a first degree relative of the other two 2. At least two successive generations affected 3. At least one of the syndrome-associated cancers should be diagnosed at <50 years of age 4. FAP should be excluded in any CRC cases5. Tumors should be verified whenever possible
**Revised Bethesda Guidelines**
Colorectal tumors from individuals should be tested for MSI in the following situations 1. CRC diagnosed in a patient who is <50 years of age 2. Presence of synchronous or metachronous CRC, or other HNPCC-associated tumors regardless of age. 3. CRC with MSI-H histology diagnosed in a patient who is <60 years of age. 4. CRC diagnosed in one or more first-degree relatives with an HNPCC-related tumor, with one of the cancers being diagnosed under age 50 years. 5. CRC diagnosed in two or more first- or second-degree relatives with HNPCC-related tumors, regardless of age.

HNPCC—Hereditary Non-polyposis Colorectal Cancer, CRC—Colorectal cancer, FAP—Familial Adenomatous Polyposis, MSI-H—Microsatellite Instability-High.

**Table 2:** Clinical characteristics.

Variable	Frequency
Gender	
Female	121 (44.0%)
Male	154 (56.0%)
Age at CRC diagnosis (median, IQR)	61.0 (54.5–65.0)
Tumor location	
Right colon	60 (21.8%)
Left colon	77 (28.0%)
Rectum	138 (50.2%)
Synchronous CRC	6
Stage (AJCC 8th edition)	
I	50 (18.2%)
II	63 (22.9%)
III	146 (53.1%)
IV	16 (5.8%)
Neoadjuvant treatment	
None	162 (58.9%)
Radiotherapy	12 (4.4%)
Chemoradiotherapy	98 (35.6%)
Chemotherapy	3 (1.1%)
Resection technique	
Right hemicolectomy	55 (20.0%)
Left hemicolectomy	13 (4.7%)
Sigmoidectomy	49 (17.8%)
Anterior rectal resection	116 (42.2%)
Abdominoperineal resection	23 (8.4%)
Total colectomy/proctocolectomy	8/3 (2.9/1.1%)
Trans-anal minimally invasive surgery	2 (0.7%)
Endoscopic	7 (2.3%)
Urgent surgery for occlusion	9 (3.3%)
Intraoperatively perforated tumor	2 (0.7%)

CRC—colorectal cancer. In case of synchronous CRC, location and staging of the more advanced neoplasia was selected to present in the table.

**Table 3:** Tumor characteristics.

Variable	Frequency
Differentiation grade	
Low-grade (G1–G2)	204 (74.2%)
High-grade (G3)	23 (8.4%)
N/A	48
Histological subtype	
Mucinous	26 (9.5%)
Signet ring	2 (0.7%)
Mucinous and signed ring	3 (1.1%)
Tubular and cribiform	2 (0.7%)
Serrated	1 (0.3%)
NOS	241 (87.6%)
Lympho-vascular invasion	69 (25.1%)
Perineural invasion	37 (13.5%)
Lymphocytic infiltrate	82 (29.8%)
Tumor budding	64 (23.3%)

N/A—not available. NOS—no other specification.

**Table 4:** Clinical and molecular characterization of cases with altered MMR status by immunohistochemical analysis.

ID	Age (years)	Gender	CRC Location at Diagnosis	CRC Stage	CRC Histopathology					AC	BC	Immunohistochemistry–Unexpressed Proteins	*BRAF V600E*	*MLH1* Promoter Hyper-Methylation	Genetic Diagnosis’ Results
					G3	LV/P	Mucinous	LI	Bd						
1	64	Male	Right colon	III	+	+	−	+	+	No	No	MLH1 and PMS2	N/A	N/A	N/A
2	61	Female	Left colon	II	−	−	−	−	−	No	Yes	MLH1 and PMS2	N/A	N/A	No mutation detected
3	29	Female	Left colon	III	N/A	+	+	+	−	No	Yes	MLH1 and PMS2	No	Yes	No mutation detected
4	39	Male	Right colon	III	−	+	−	+	−	No	Yes	MLH1 and PMS2	N/A	N/A	No mutation detected
5	54	Female	Right colon	II	+	+	−	−	−	No	No	MLH1 and PMS2	No	Yes	No mutation detected
6	32	Male	Right colon	III	−	−	−	−	−	No	Yes	MSH2 and MSH6	N/A	N/A	LS-*MSH2* Frameshift mutation c.388_389delp.Gln130ValfsTer2
7	66	Male	Rectum	III	−	−	−	+	−	No	No	PMS2	N/A	N/A	N/A
8	60	Female	Rectum	III	−	−	−	−	−	No	No	MLH1 and PMS2	N/A	N/A	N/A
9	67	Female	Right colon	III	+	−	−	+	−	No	No	MLH1 and PMS2	No	Yes	N/A
10	63	Male	Rectum	III	−	−	−	+	−	No	No	MSH6	N/A	N/A	N/A
11	64	Male	Rectum	III	−	−	−	−	−	No	Yes	MLH1 and PMS2	N/A	N/A	N/A
12	62	Male	Left colon	I	−	−	−	−	−	No	No	MSH6	N/A	N/A	N/A
13	65	Male	Left colon	I	−	−	−	−	−	No	Yes	MSH2	N/A	N/A	N/A
14	51	Male	Right colon	II	−	+	−	+	−	No	Yes	MLH1 and PMS2	N/A	N/A	N/A
15	54	Male	Right colon	II	−	−	−	+	−	No	Yes	MLH1 and PMS2	No	No	No mutation detected
16	67	Male	Right colon	II	−	−	−	−	−	No	No	MLH1 and PMS2	N/A	N/A	N/A
17	67	Male	Right colon	II	−	−	−	+	−	No	No	MLH1 and PMS2	N/A	N/A	No mutation detected
18	67	Male	Right colon	III	+	+	−	+	+	No	Yes	PMS2	Yes	N/A	N/A
19	67	Male	Right colon	IV	+	+	−	+	+	No	Yes	PMS2	Yes	N/A	N/A
20	63	Male	Right colon	II	−	−	−	+	−	No	No	MLH1 and PMS2	Yes	N/A	N/A
21	56	Male	Rectum	IV	−	+	−	−	+	No	Yes	MLH1 and PMS2	N/A	N/A	No mutation detected
22	42	Male	Right colon	I	−	−	−	−	−	No	Yes	MLH1 and PMS2	No	No	LS-*MLH1* Missense mutation c.2041G > Ap.(Ala681Thr)
23	55	Male	Right colon	III	−	−	−	−	+	No	No	PMS2	No	N/A	LS-*PMS2* Deletion exons 1 to 14 (c.(?-87)_(2445+1_2446-1)del)
24	64	Male	Right colon	IV	+	+	−	−	+	No	Yes	MLH1, PMS2 and MSH6	No	No	No mutation detected
25	26	Male	Rectum	III	−	−	−	−	−	No	Yes	PMS2 N/A (artifacts), MLH1, MSH2 and MSH6 expressed	No	N/A	LS-*PMS2* Deletion exons 12 to 14 (c.(2006+1_2007-1)_(2445+1_2446-1)del)

ID–identification; CRC–colorectal cancer; G3–poorly differentiated; LV/P–lympho-vascular and/or perineural invasion; Bd–Budding; LI–lymphocytic infiltrate; AC–Amsterdam criteria; BC–Revised Bethesda criteria; N/A–non applicable/ non available; LS–Lynch Syndrome.

## Data Availability

Data not shared due to confidentiality.
